# The Circulating Cholangiocarcinoma Protein Biomarkers CRP and MASP2 Also Predict Gallbladder Cancer Risk

**DOI:** 10.1002/ijc.70661

**Published:** 2026-07-19

**Authors:** Linda Zollner, Jeroen Krijgsveld, Dominique Scherer, Justo Lorenzo Bermejo, the EULAT Eradicate GBC Consortium

**Affiliations:** ^1^ Statistical Genetics Research Group, Institute of Medical Biometry Heidelberg University Heidelberg Germany; ^2^ Division of Proteomics of Stem Cells and Cancer German Cancer Research Center Heidelberg Germany; ^3^ Heidelberg Medical Faculty Heidelberg University Heidelberg Germany; ^4^ Laboratory of Biostatistics for Precision Oncology Institut de cancérologie Strasbourg Europe Strasbourg France

**Keywords:** cholangiocarcinoma, circulating biomarkers, gallbladder cancer, proteomics, risk prediction

## Abstract

In a recent publication, Lapitz et al. reported differences in serum levels that predict the development of cholangiocarcinoma (CCA) in patients with primary sclerosing cholangitis prior to clinical manifestation. We examined whether these biomarkers also predict the risk of gallbladder cancer (GBC) in European prospective plasma samples from 24 GBC cases and 90 control individuals. After logarithmic transformation and quantile normalisation of individual protein levels measured with a timsTOF PRO mass spectrometer, we fitted univariate logistic regression models and applied backward model selection to identify the optimal model for GBC risk prediction. CRP and MASP2, previously reported markers of CCA, were found to be predictive for GBC risk as well (*p* value < 0.05), and complemented by age at blood sampling, provided an area under the receiver operating characteristic curve of 0.80 (95% confidence interval 0.69–0.92) when combined in a prediction model for GBC risk. We further examined the mRNA expression of CRP and MASP2 in serum samples from 82 GBC cases and 79 control subjects from Chile. CRP mRNA levels were elevated in Chilean GBC cases, but MASP2 showed an opposite trend. While there are considerable differences in the design of the discovery study and ours, the finding that circulating levels of CRP and MASP2 are associated with the risk of both CCA and GBC in Europeans highlights the need for further research into potential shared mechanisms and strategies for the prevention of these two aggressive biliary tumours.

## What's New?

Like cholangiocarcinoma, gallbladder cancer lacks liquid biopsy biomarkers for screening, especially in persons at increased risk, such as patients with primary sclerosing cholangitis or gallstone disease. This prospective study demonstrates that the circulating levels of CRP and MASP2, initially associated with the risk of cholangiocarcinoma in patients with primary sclerosing cholangitis, also predict the risk of gallbladder cancer. The findings point to potential mechanisms shared by the two types of cancer and the possibility of simultaneous prevention for these two aggressive biliary tumours.

## Graphical abstract

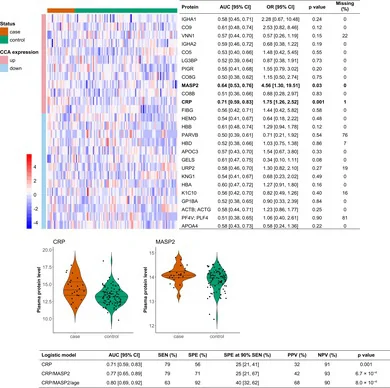

**Abbreviations**
AUC: area under receiver operating characteristic curve
BBMRI‐LPC: Biobanking and Biomolecular Resources Research Infrastructure—Large Prospective Cohorts
CCA: cholangiocarcinoma
CI: confidence interval
EPIC: European Prospective Investigation into Cancer and Nutrition
ESTHER: Early Detection and Optimised Therapy of Chronic Diseases in the Elderly Population study
EULAT Eradicate GBC Explanation: European‐Latin American Research Consortium towards Eradication of Preventable Gallbladder Cancer
GBC: gallbladder cancer
NPV: negative predictive value
OR: odds ratio
PPV: positive predictive value
PSC: primary sclerosing cholangitis
ROC: receiver operating characteristic
SEN: sensitivity
SPE: specificity

## 1 Introduction

In the article ‘Liquid biopsy‐based protein biomarkers for risk prediction, early diagnosis, and prognostication of cholangiocarcinoma’, Lapitz et al. [1] identified protein levels in serum extracellular vesicles and whole serum associated with the risk of cholangiocarcinoma (CCA). The authors identified 32 circulating proteins associated with CCA risk in patients with primary sclerosing cholangitis (PSC) and proposed a model based on the serum levels of CRP, FIBRINOGEN, PIGR and FRIL to predict CCA risk in PSC patients before the onset of clinical signs of malignancy.

Gallbladder cancer (GBC) is another highly aggressive biliary tumour that shows wide geographic and ethnic variability. It is relatively rare in most high‐income countries, but high incidence and mortality rates are reported in Latin America, and East and South Asia. Similar to CCA, GBC shows no or only non‐specific symptoms until the late stages of the disease, making the tumour highly lethal and underscoring the urgent need of early detection biomarkers [2, 3, 4]. As with CCA, GBC lacks liquid biopsy biomarkers for screening, especially in persons at increased risk, such as patients with PSC or gallstone disease [3, 5]. Here, we have examined whether the identified CCA protein biomarkers also predict GBC risk in the prospective European samples collected within the European‐Latin American Research Consortium towards Eradication of Preventable Gallbladder Cancer (EULAT Eradicate GBC) [6], and analysed Chilean mRNA expression data.

## 2 Materials and Methods

EULAT Eradicate GBC is a European–Latin American research consortium whose goal is to identify environmental, lifestyle, genetic and molecular differences in GBC risk, and to translate these differences into GBC prevention programmes [6]. So far, data and samples from over 200 prospective GBC case–control pairs nested within large European prospective cohorts, covering Western European countries with a low GBC incidence, have been collected as part of the ‘Biobanking and Biomolecular Resources Research Infrastructure—Large Prospective Cohorts (BBMRI‐LPC)’ project, and over 14,000 study participants with gallstones and GBC have been recruited in high‐incidence regions of South America (northeastern Argentina, Bolivia, Chile and southern Peru).

The European population investigated in this study comprised 24 GBC cases and 90 control persons from the Estonian Genome Project (*n* = 9), the Early Detection and Optimised Therapy of Chronic Diseases in the Elderly Population (ESTHER) study (*n* = 6) and the European Prospective Investigation into Cancer and Nutrition (EPIC) (*n* = 99) study [7, 8]. The case group comprised exclusively individuals who developed GBC (ICD‐10: C23), with a maximum time between blood sampling and GBC diagnosis of 5 years. Data on abdominal sonography were not available to identify gallstone patients in the control group, but the proportion of controls affected by gallstone disease is likely to be representative of the corresponding proportion in the cohorts that gave rise to the cases. Among GBC cases, 75% were female, with a mean age at recruitment of 63 years (95% CI: 51–82 years). In the control group, 79% were female, and the mean age was 59 years (95% CI: 43–75 years). Among the cases, 42% of individuals had a body mass index of > 30, compared with 20% in the control group.

EDTA plasma samples were prepared for proteome analysis on an automated platform using the SP3 procedure for protein clean‐up and digestion [9]. Peptides were then analysed by LC–MS using an EASY‐nLC chromatography system coupled to a timsTOF PRO mass spectrometer. Raw data were processed with DIA‐NN software [10]. Of the 32 serum proteins that predict CCA risk according to Lapitz et al. [1], 26 were detected in the plasma samples examined in this study.

After logarithmic transformation of all individual protein levels measured in our study, missing values for the 26 candidate proteins were replaced by the median values of the remaining samples. All other protein groups with missing values were excluded from further analysis. The protein levels of the remaining 240 protein groups were then quantile normalised, first for cases only, then for controls only and finally for cases and controls together. We then fitted univariate logistic regression models and the corresponding receiver operating characteristic (ROC) curves for each of the 26 candidate proteins to assess the association between protein levels and GBC status by estimating the odds ratio (OR) with a corresponding 95% confidence interval (CI) and the area under the ROC curve (AUC). Subsequently, we applied backward model selection on proteins with a *p* value < 0.2, considering age at blood sampling as an additional covariate to identify the optimal model for GBC risk prediction. We calculated the AUC, as well as the sensitivity, specificity, positive predictive value (PPV) and negative predictive value (NPV) for the optimal cut‐off point, as determined using the Youden index. We also calculated the specificity of the logistic model for a sensitivity of 90% using bootstrapping. To do this, we randomly selected 90% of the cases and 90% of the controls from the study sample 1000 times, performed the corresponding ROC analysis and extracted the median and 95% CIs from the bootstrap iterations.

To further investigate the predictive value of CRP and MASP2 in a region with a high GBC incidence, we examined their mRNA expression in serum samples from 161 Chilean participants recruited by the EULAT Eradicate GBC consortium. The case group comprised 82 individuals diagnosed with GBC, 70% of whom were female and whose mean age was 62 years (95% CI: 40–81 years). The control group comprised 79 individuals who underwent cholecystectomy for the treatment of gallstone disease, with a mean age of 61 years (95% CI: 41–79 years), 67% of whom were female. The protocol used for small RNA extraction and sequencing has been described previously [11, 12, 13].

As with the European plasma protein data, Chilean serum mRNA data were also subjected to a logarithmic transformation. mRNAs with > 15% zero counts were excluded from further analysis, while CRP and MASP2 were retained. Following quantile normalisation, differences in mRNA expression were assessed and visualised using violin plots. Individual proportions of genetic ancestry were estimated using the ADMIXTURE software as previously described [14, 15], and separated analyses were conducted for individuals with a proportion of Indigenous American Mapuche ancestry below and above the median, which was 34.3%.

## 3 Results

**FIGURE 1:**
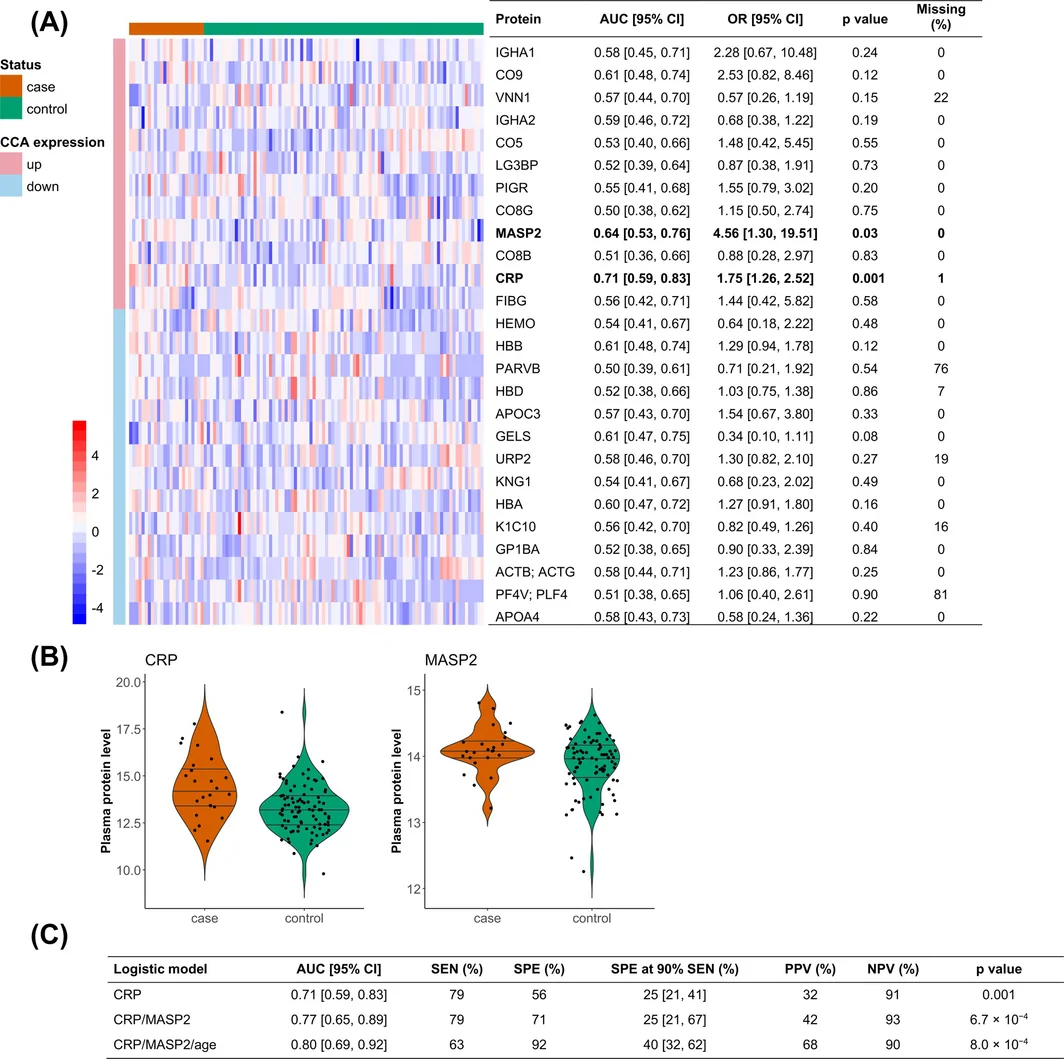
(A) Heat map of protein expression levels in 24 European GBC cases (orange bar) and 90 European controls (green bar), univariate logistic regression results and proportion of plasma samples with missing protein data. Proteins associated with a higher CCA risk are marked with a pink vertical bar, and those associated with a lower CCA risk with a blue bar. (B) Violin plots for plasma protein expression of CRP and MASP2, included together with age at blood sampling in the best prediction model for GBC based on backward selection. (C) Results of univariate and multiple logistic regression models. AUC, area under receiver operating characteristic curve; CCA, cholangiocarcinoma; CI, confidence interval; GBC, gallbladder cancer; NPV, negative predictive value; OR, odds ratio; PPV, positive predictive value; SEN, sensitivity; SPE, specificity.

Proteomic analysis identified 361 protein groups in the prospective EDTA plasma samples from 24 GBC cases and 90 control subjects. Figure 1A depicts the levels of proteins predictive of CCA in the investigated prospective samples. A heat map is shown on the left, and logistic regression and ROC analysis results are shown on the right, together with the percentage of samples with missing values. The levels of two proteins predictive of CCA (MASP2 and CRP) were also predictors of GBC risk in the prospective plasma samples investigated (*p* value < 0.05). Figure 1B shows the violin plots for the two proteins, showing that increased protein levels are associated with a higher risk both of CCA and GBC. Backward selection on CO9, VNN1, IGHA2, PIGR, MASP2, CRP, HBB, GELS, HBA and age at blood sampling identified a logistic regression model with CRP, MASP2 and age at blood sampling as the best model, resulting in an AUC of 0.80, 95% CI 0.69–0.92 and specificity of 40% (95% CI 32%–62%) for sensitivity of 90% (Figure 1C).

**FIGURE 2:**
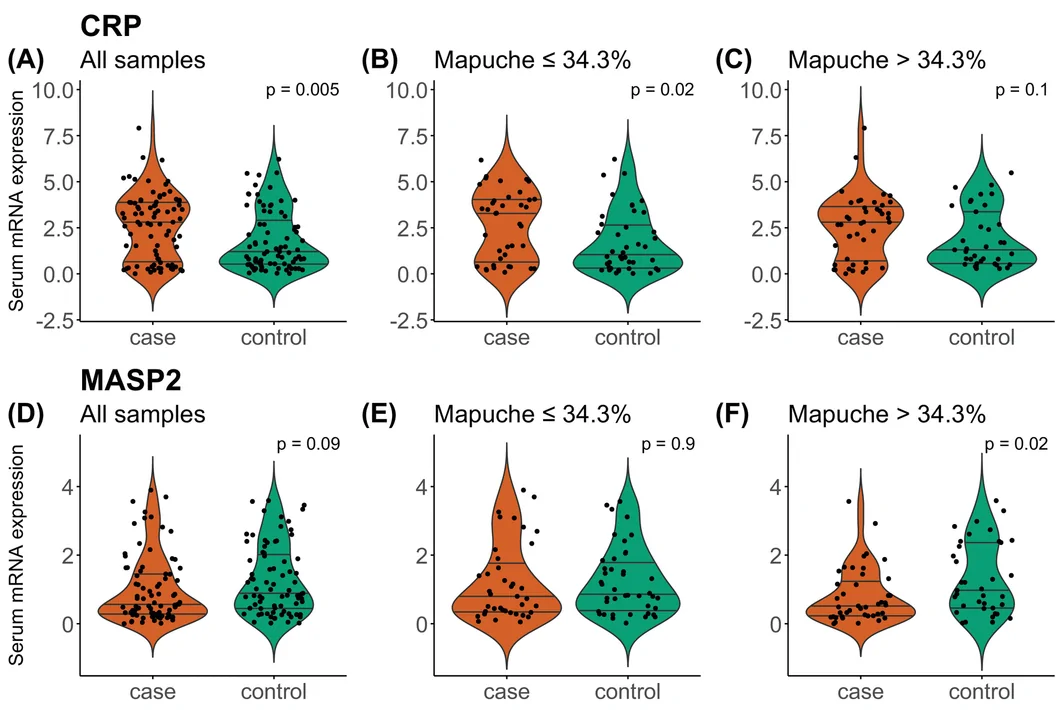
Violin plots illustrating mRNA expression in serum samples from 82 Chilean GBC and 79 Chilean gallstone disease patients. The top row (A–C) displays results for CRP, while the bottom row (D–F) shows results for MASP2. The first column (A, D) shows mRNA expression for all samples. The middle and right columns show mRNA levels stratified by the individual proportion of Mapuche ancestry, with individuals having a Mapuche proportion of 34.3% or less (corresponding to the sample median) shown in (B, E), and those with a Mapuche ancestry proportion above 34.3% in (C, F). All plots include *p* values from a univariate regression model fitted to the respective dataset.

Figure 2 shows the serum mRNA expression of CRP and MASP2 in the Chilean patients with GBC and gallstone disease. In line with the findings of Lapitz et al. and our analysis of European plasma proteomics data, serum CRP mRNA expression was increased in GBC cases (*p* value = 0.005, Figure 2A). Stratified analyses according to Indigenous Mapuche ancestry (Figure 2B,C) confirmed the overexpression in GBC cases, particularly in individuals with a Mapuche proportion below 34.3% (*p* value = 0.02, Figure 2B). However, in contrast to the results of Lapitz et al. and our plasma proteomics data, MASP2 showed lower serum mRNA expression levels in Chilean GBC cases (*p* value = 0.09, Figure 2D). When stratified by Indigenous Mapuche ancestry (Figure 2E,F), the difference in serum mRNA expression between GBC cases and gallstone disease controls was larger in individuals with a Mapuche proportion of over 34.3% (*p* value = 0.02, Figure 2F).

## 4 Discussion

In a panel of proteins previously identified as indicators of CCA risk, we also identified CRP and MASP2 as predictors of GBC risk in European individuals, and confirmed the increased CRP levels in Chilean GBC patients. The fact that circulating levels of these two proteins predict risk for both CCA and GBC in Europeans suggests that these cancers share inflammatory and immune‐related mechanisms, supporting the notion that tumours of the biliary tract may have overlapping pathways of early development. If validated in independent studies, CRP and MASP2 could be incorporated into blood tests to identify individuals at elevated risk of GBC for ultrasound or imaging surveillance before symptoms appear. These tests would be particularly useful in regions with high GBC incidence (e.g., parts of Latin America and Asia). From a methodological perspective, this study demonstrates that mass‐spectrometry–based proteomics, followed by normalisation and logistic modelling, can identify predictive biomarkers in modestly sized prospective datasets, and that it is possible to repurpose risk biomarkers across related cancers, thereby reducing duplicate research and accelerating the development of novel screening strategies. We show the feasibility of testing markers discovered in one setting (PSC → CCA) in a different population (general population at risk of GBC). The results justify larger, multicentre validation studies using well‐annotated cohorts such as the EULAT Eradicate GBC consortium.

There are some key differences between the study by Lapitz et al. [1] and our analyses. While they explored CCA risk prediction, early detection and prognosis, we confined ourselves to GBC risk prediction using prospective samples. Some of the CCA protein marker candidates, including FIBRINOGEN, PIGR and FRIL, showed *p* values for association with GBC risk higher or equal to 0.2 and they were not considered in our backward model selection. In addition, Lapitz et al. measured proteins in serum extracellular vesicles and whole serum, while we primarily analysed plasma samples, which may reflect distinct aetiological components. GBC is less common than CCA in Europe and our sample size was smaller, which limits statistical power, but we leveraged the results of Lapitz et al. to investigate a smaller set of biomarkers prioritising the most relevant targets. In summary, circulating levels of CRP and MASP2 predict both CCA and GBC risk, opening the possibility for further studies to assess shared mechanisms and simultaneous prevention of these two aggressive biliary tumours.

## Author Contributions

**Linda Zollner:** formal analysis, investigation, visualization, writing – original draft, writing – review and editing. **Jeroen Krijgsveld:** data curation, supervision, writing – review and editing, funding acquisition. **Dominique Scherer:** writing – review and editing, funding acquisition. **Justo Lorenzo Bermejo:** supervision, writing – review and editing, conceptualization, project administration, methodology, funding acquisition.

## Funding

This study was supported by the European Union's Horizon 2020 research and innovation program (grant 825741), the European Union's FP7 project ‘Biobanking and Biomolecular Research Infrastructure ― Large Prospective Cohorts’ (grant 313010) and state funds approved by the State Parliament of Baden‐Württemberg for the Innovation Campus Health + Life Science alliance Heidelberg Mannheim.

## Ethics Statement

The plasma samples analysed were provided by the following large European prospective cohorts participating in the collaborative study ‘Identification of biomarkers for gallbladder cancer risk prediction—Towards personalized prevention of an orphan disease’ as part of the ‘Biobanking and Biomolecular Resources Research Infrastructure—Large Prospective Cohorts (BBMRI‐LPC)’ project: the Estonian Genome Project (approval 233/T‐11), the Early Detection and Optimised Therapy of Chronic Diseases in the Elderly Population (ESTHER) study (project 58/2000 Heidelberg Medical Faculty) and the European Prospective Investigation into Cancer and Nutrition (EPIC) study (project 16‐23). The investigation of Chilean patients was approved by the ethics committees of Servicio de Salud Metropolitano Oriente, Servicio de Salud Metropolitano Sur Oriente and Servicio de Salud Metropolitano Central in Santiago de Chile, Servicio de Salud Maule in Talca, Chile, Universidad Católica del Maule in Talca, Chile, Servicio de Salud Concepción in Concepción, Chile, Servicio de Salud Araucanía Sur in Temuco, Chile, Unidad de Investigación Hospital San Juan de Dios in Santiago de Chile and the Medical Faculties of Universidad de Chile and Pontificia Universidad Católica de Chile.

## Conflicts of Interest

The authors declare no conflicts of interest.

## Data Availability

The data that support the findings of this study are available from the corresponding author upon reasonable request.
